# Specific Features of the Hypothalamic Leptin Signaling Response to Cold Exposure Are Reflected in Peripheral Blood Mononuclear Cells in Rats and Ferrets

**DOI:** 10.3389/fphys.2017.00581

**Published:** 2017-08-15

**Authors:** Bàrbara Reynés, Marlou Klein Hazebroek, Estefanía García-Ruiz, Jaap Keijer, Paula Oliver, Andreu Palou

**Affiliations:** ^1^Laboratory of Molecular Biology, Nutrition, and Biotechnology, Universitat de les Illes Balears Palma, Spain; ^2^CIBER de Fisiopatología de la Obesidad y Nutrición (CIBERobn) Palma, Spain; ^3^Balearic Islands Health Research Institute (IdISBa) Palma, Spain; ^4^Human and Animal Physiology Group, Wageningen University and Research Centre Wageningen, Netherlands

**Keywords:** cold exposure, hypothalamus, food intake, biomarkers, leptin

## Abstract

**Objectives:** Cold exposure induces hyperphagia to counteract fat loss related to lipid mobilization and thermogenic activation. The aim of this study was investigate on the molecular mechanisms involved in cold-induced compensatory hyperphagia.

**Methods:** We analyzed the effect of cold exposure on gene expression of orexigenic and anorexigenic peptides, and of leptin signaling-related genes in the hypothalamus of rats at different ages (1, 2, 4, and 6 months), as well as in ferrets. We also evaluated the potential of peripheral blood mononuclear cells to reflect hypothalamic molecular responses.

**Results:** As expected, cold exposure induced hypoleptinemia in rats, which could be responsible for the increased ratio of orexigenic/anorexigenic peptides gene expression in the hypothalamus, mainly due to decreased anorexigenic gene expression, especially in young animals. In ferrets, which resemble humans more closely, cold exposure induced greater changes in hypothalamic mRNA levels of orexigenic genes. Despite the key role of leptin in food intake control, the effect of cold exposure on the expression of key hypothalamic leptin signaling cascade genes is not clear. In our study, cold exposure seemed to affect leptin signaling in 4-month-old rats (increased *Socs3* and *Lepr* expression), likely associated with the smaller-increase in food intake and decreased body weight observed at this particular age. Similarly, cold exposed ferrets showed greater hypothalamic *Socs3* and *Stat3* gene expression. Interestingly, peripheral blood mononuclear cells (PBMC) mimicked the hypothalamic increase in *Lepr* and *Socs3* observed in 4-month-old rats, and the increased *Socs3* mRNA expression observed in ferrets in response to cold exposure.

**Conclusions:** The most outstanding result of our study is that PBMC reflected the specific modulation of leptin signaling observed in both animal models, rats and ferrets, which points forwards PBMC as easily obtainable biological material to be considered as a potential surrogate tissue to perform further studies on the regulation of hypothalamic leptin signaling in response to cold exposure.

## Introduction

Cold exposure elicits a range of physiological effects in overall body processes in order to maintain body temperature (Kizaki et al., 1996; Sonna et al., 2002; Cannon and Nedergaard, 2004; Karp, 2012). Notably, in small mammals, cold exposure induces non-shivering thermogenesis is brown adipose tissue (BAT) and browning white adipose tissue (WAT), which results in fat mobilization and weight loss (Himms-Hagen, 1990; Palou et al., 1998; Nedergaard et al., 2007; Fuster et al., 2009; Reynés et al., 2015). Cold exposure and its resulting fat loss produce severe hypoleptinemia, which is accompanied by increased compensatory hyperphagia (Bing et al., 1998a; Lau et al., 2016).

The key regulator of food intake control is the hypothalamus, particularly the arcuate nucleus, which plays a crucial role in the integration and coordination of peripheral and central orexigenic and anorexigenic signals (Sahu, 2004; Palou et al., 2009). Briefly, leptin and other satiety signals inhibit the orexigenic neurons that produce agouti related peptide (AgRP) and neuropeptide Y (NPY), which are key enhancers of appetite (Morton and Schwartz, 2001; Valassi et al., 2008). Orexigenic signals such as ghrelin, or the absence of insulin and leptin, induce the contrary response (Ellacott and Cone, 2004). Accordingly, some studies revealed that *Agrp* gene expression increases due to the low leptin levels related to cold exposure (Tang et al., 2009; Lau et al., 2016). Nevertheless, in spite of the hypoleptinemia resulting from cold exposure, *Npy* regulation by cold is controversial (Mercer et al., 1997; Bing et al., 1998a,b; Park et al., 2007; Tang et al., 2009; Lau et al., 2016), since besides its orexigenic role, it also acts as a hypothermic peptide with potent central anti-thermogenic actions (Egawa et al., 1991; Billington et al., 1994). At the other end of the scale, the anorexigenic signals induce the production of pro-opiomelanocortin (POMC) and cocaine and amphetamine regulated transcript peptide (CART) in other neuron populations, also located in the arcuate nucleus, which induce satiety (Seeley and Woods, 2003; Porte et al., 2005; Valassi et al., 2008). While there is no evidence regarding the regulation of POMC by cold, it has been shown that CART signaling is required for maintenance of energy homeostasis under cold exposure (Lau et al., 2016). The long form of the leptin receptor (LEPR/ObRb) and the suppressor of cytokine signaling 3 (SOCS3), expressed in arcuate hypothalamic neurons, are commonly regarded as key hypothalamic factors mediating leptin regulation of feeding control (Blundell et al., 2001). However, the effects of cold exposure on leptin signal transduction are not fully elucidated, since one study indicates increased hypothalamic *Lepr* gene expression after cold exposure (Mercer et al., 1997) while another shows no changes in hypothalamic leptin transduction, suggesting that leptin sensitivity remains unchanged during cold acclimation (Tang et al., 2009).

Aging is a process with an important impact on the metabolism, including cold response. In rodents, BAT thermogenesis activation by cold exposure decreases with age (Reynés et al., 2015). Accordingly, in rabbits, the responses of sympathetic neurons to cold stress have been described to be reduced (Andrews et al., 1993). This suggests that food intake control in response to cold could be affected by aging. Thus, we considered it useful to study the effects of cold exposure on neuronal hypothalamic regulation at different ages of rat development, paying attention to the effects upon leptin signaling.

Neuronal hypothalamic regulation of the cold induced increase in food intake is difficult to study in humans due to inaccessibility of hypothalamic samples. Rodents provide a realistic alternative, but in rodents, in contrast to humans, BAT thermogenesis is a major contributor to cold induced energy expenditure, which is thought to induce the hyperphagic response. Ferrets (*Mustela putorius furo*) provide an alternative animal model, closer to humans in terms of adipose tissue organization (Murano et al., 2005; Liew et al., 2006; Wolfgang et al., 2006) as well as the thermogenic contribution of BAT to energy expenditure in response to cold exposure (Fuster et al., 2009).

In summary, the effects of cold exposure on the hypothalamic leptin signaling cascade, and on the regulation of food intake are not clear and may be affected by age. Studies in rats should be complemented by studies in other species, due to the different role of BAT in cold induced energy expenditure in rats compared to humans, in order to strengthen the data. Ideally, the responses should be analyzed in humans, but the hypothalamus is inaccessible for molecular analysis. Therefore, it is worthwhile to examine whether hypothalamic responses are reflected by responses in other tissues that are more easily accessible and amenable to molecular analyses, such as blood cells. Our previous work shows that peripheral blood mononuclear cells (PBMC) can be a surrogate tissue to study metabolic responses of internal tissues, including hypothalamus (Caimari et al., 2010b; Reynés et al., 2014, 2015), leading us to examine whether PBMC reflect hypothalamic adaptations to cold exposure.

Thus, to better understand the hypothalamic response to cold exposure, we analyzed molecular satiety and leptin signaling gene expression in rats of different ages, validated these responses in ferrets, and examined the validity of PBMC as a surrogate tissue.

## Materials and methods

### Animals and experimental design

All animal experimental procedures have been reviewed and approved by the Bioethical Committee of the University of the Balearic Islands and the guidelines for the use and care of laboratory animals of the university were followed. We performed experiments with two different animal species: rats and ferrets. Both cohorts of animals used in this study were also used for other experimental purposes (Reynés et al., 2015, 2017). In the rat experiment, female Wistar Rats (Charles River Laboratories España, SA, Barcelona, Spain) of different ages (1, 2, 4, and 6 month) were distributed into a control or a cold group (each *n* = 5–6). The control group was acclimatized to room temperature (22 ± 2°C). We selected 22°C instead of thermoneutrality (30°C) because it is an equivalent temperature to mimic human physiology (Speakman and Keijer, 2012). The cold group was acclimatized to 4°C for 1 week. The cold-exposed animals were housed individually with sawdust as a nesting material, with a 12:12 h light/dark cycle, and had free access to a standard chow diet on cage tops (Panlab, Barcelona, Spain). Food intake was recorded twice a week in Control and Cold groups. Animals were weighed before and after cold exposure. Animals were sacrificed by decapitation and the hypothalamus was rapidly removed, frozen in liquid nitrogen and stored at −80°C for RNA analysis. Different WAT depots (inguinal, retroperitoneal, epididymal, and mesenteric) were also excised and weighed to calculate the adiposity of each rat, which was considered as the sum of these depots expressed as a percentage of total body weight. Part of troncular blood was collected from the neck in corning tubes, stored at room temperature for 1 h, and then centrifuged at 1,000 g for 10 min at 4°C to collect serum; this serum was used to analyze circulating parameters. In the ferret experiment, 3-month-old male ferrets, *Mustela Putorius Furo*, (Cunipic, Lleida, Spain), were divided into two groups (*n* = 6–7): a Control group, acclimatized to room temperature (22 ± 2°C), and a Cold group, acclimatized to 4°C for 1 week. The cold-exposed animals were housed individually. All animals were exposed to a light/dark cycle of 12 h and had free access to water and diet (Gonzalo Zaragoza Manresa SL, Alicante, Spain). In all ferret cages, we used cat litter as a nesting material. Animals were weighed before and after cold exposure. Sacrifice was performed by exsanguination of ferrets anesthetized with10 mg/kg of ketamine hydrochloride (Imalgène 1000, Merial Laboratorios SA, Lyon, France) and 80 mg/kg medetomidine (Domtor, Orion Pharma, Espoo, Finland). Afterwards, the hypothalamus was rapidly collected and frozen in liquid nitrogen and stored at −80°C until transcriptomic analysis. Moreover, different visceral and subcutaneous adipose depots (interscapular, inguinal, retroperitoneal and mesenteric) were obtained and weighed.

### Measurement of circulating parameters (leptin and ghrelin) in rats

Serum leptin levels were determined using an enzyme-linked immunosorbent assay kit (R&D Systems, Minneapolis, MN, USA). Serum ghrelin levels were determined using a ghrelin enzyme immunosorbent assay kit (Phoenix Europe, Karlsruhe, Germany). Leptin and ghrelin levels were not measured in ferrets due to the lack of diagnostic kits for this animal model.

### Isolation of peripheral blood mononuclear cells

In rats, part of the troncular blood samples from Control and Cold animals at different ages, collected in Corning tubes with EDTA 0.5 M as an anticoagulant, was used to isolate PBMC by OptiPrep gradient separation (Sigma-Aldrich Química, SL, Madrid, Spain) as previously described (Reynés et al., 2015). In the ferret experiment, PBMC were isolated from blood samples collected using heparin in NaCl (0.9%) as anticoagulant by Ficoll gradient separation according to the manufacturer's instructions (GE Healthcare Bio Sciences, Barcelona, Spain), with some modifications (Reynés et al., 2017).

### Total RNA isolation

Total RNA was isolated from rat and ferret hypothalamus and PBMC using Tripure Reagent (Roche Diagnostics Barcelona, Spain). Total RNA from ferret and rat hypothalamus was purified by precipitation with 3 M sodium acetate and absolute ethanol, while ferret and rat PBMC samples were purified with E.Z.N.A. MicroElute RNA Clean Up (Omega Bio-Tek, Vermont, USA); additionally, ferret PBMC samples were also precipitated with sodium acetate. NanoDrop ND 1000 spectrophotometer (NanoDrop Technologies, Wilmington, DE, USA) was used to quantify RNA yield and its integrity was confirmed using agarose gel electrophoresis.

### Quantitative real time reverse transcriptase polymerase chain reaction (qRT-PCR) analysis

Expression of genes of interest was determined by qRT-PCR. 250 ng of total RNA from the hypothalamus and 50 ng from PBMC were transcribed to cDNA. PCR from diluted (1/10 for hypothalamus and 1/5 for PBMC) cDNA samples was performed as previously described (Reynés et al., 2015). The threshold cycle (Ct) was calculated using StepOne Software v2.0 (Applied Biosystems). The relative expression of each mRNA was calculated as a percentage of control rats using the 2^−ΔΔCt^ method (Livak and Schmittgen, 2001). For rat samples, hypothalamus data were normalized against *Gdi1* (*Guanosine diphosphate dissociation inhibitor 1*), used as a reference gene based on previous studies (Reynés et al., 2014), and PBMC data against *Lrp10* (*Low-density lipoprotein receptor-related protein 10*), which has been shown to be a useful reference gene for cold exposure studies (Gabrielsson et al., 2005; Caimari et al., 2012; Reynés et al., 2015). For ferret samples, *Rgp1* (*Homolog, RAB6A GEF complex partner 1*) was chosen as the housekeeping gene for hypothalamus data and *Mettl2a* (*Methyltransferase-like protein 2A*) for PBMC data, both selected as stable genes based on a microarray study (unpublished data). Primers for the different genes are described in Table 1 and were obtained from Sigma Genosys (Sigma Al-drich Química SA, Madrid, Spain).

**Table 1:** Nucleotide sequences of primers used for qRT-PCR amplification and size of the PCR products for rats and ferrets.

**Gene**	**Forward primer (5′−3′)**	**Reverse primer (5′−3′)**	**Amplicon size (bp)**
**WISTAR RATS**			
*Agrp*	AGAGTTCTCAGGTCTAAGTCT	CTTGAAGAAGCGGCAGTAGCACGT	210
*Cart*	AGAAGAAGTACGGCCAAGTCC	CACACAGCTTCCCGATCC	84
*Lepr*	AGCCAAACAAAAGCACCATT	TCCTGAGCCATCCAGTCTCT	174
*Npy*	TGGACTGACCCTCGCTCTAT	GTGTCTCAGGGCTGGATCTC	188
*Pomc*	CCTGTGAAGGTGTACCCCAATGTC	CACGTTCTTGATGATGGCGTTC	266
*Socs3*	ACTGAGCCGACCTCTCTCCT	CCCCTCTGACCCTTTCTTTG	172
*Stat3*	GCTGACCAATAACCCCAAGA	ACACCCTGAGTAGTTCACACCA	181
*Lrp10 (reference)*	TCCCCTTTCTTCTCCTCCTC	TTACCGTCTGTTCCTTGCTG	198
*Gdi1 (reference)*	CCGCACAAGGCAAATACATC	GACTCTCTGAACCGTCATCAA	159
**FERRETS**			
*Agrp*	GCAGAACAGGCAGAAGA	TTGAAGAAGCGGCAGTAGC	176
*Cart*	AGAAGAAGTACGGCCAAGTCC	CACACAGCTTCCCGATCC	84
*Lepr*	TTGCCATTTGCAGAGAGATG	TGCTTTCACACTGGATGGAG	175
*Npy*	ACCAGGCAGAGGTATGGAA	AACCGCAGAGTTGAGCAC	235
*Pomc*	GCCTACAAGATGGAGCACTT	TTGAACAGCGTCACCAAGG	108
Socs3	CCCAGGAGAGCCTATTACATC	GTCTTCCGACAGACAGAGATGCTG	107
*Stat3*	GCTGACCAATAACCCCAAGA	ACACCCTGAGTAGTTCACACCA	181
*Mettl2a (reference)*	GGTCGTTCCAGACAAGATGC	CCGTCCCCTCTCACATAGAA	162
*Rgp1 (reference)*	TCGGGGTCAGTCAGTCAAGT	CCTTCTTCCCACCTTCATCC	191

*Agrp, agouti−related peptide; Cart, Cocaine and amphetamine regulated transcript; Lepr, leptin receptor; Npy, neuropeptide Y; Pomc, Proopiomelanocortin; Socs3, suppressor of cytokine signaling 3; Stat3, signal transducer and activator of transcription 3; Lrp10, low−density lipoprotein receptor−related protein 10; Gdi1, guanosine diphosphate dissociation inhibitor 1; Mettl2a, methyltransferase−like protein 2A; Rgp1, homolog, RAB6A GEF complex partner 1*.

### Statistical analysis

All data are expressed as the mean ± SEM. ANOVA was used to analyze differences between ages, using least significant difference (LSD) *post-hoc* comparisons. The effect of cold exposure was analyzed using a Student's *t*-test. To compare between two groups, data were checked for normality using the Shapiro-Wilks test. Analyses were performed with SPSS for Windows (SPSS, Chicago, IL, USA). A Pearson correlation analysis was also performed for the parameters studied. Threshold of significance was defined at *p* < 0.05, and is indicated when different.

## Results

### Animal and serum parameters

Body weight and adiposity data of the same cohorts of rats and of ferrets have been previously published (Reynés et al., 2015, 2017) and are shown, together with additional parameters, in Table 2. Exposure of rats to 4°C for 1 week decreased adiposity at the different ages studied (1, 2, 4, and 6 months), although this was translated into lower body weight only at the age of 1 and 4 months (Table 2A). Decreased adiposity in cold-exposed animals was accompanied by decreased circulating leptin levels at all ages. On the other hand, cold exposure induced increased food intake at all the ages studied, but to a lesser extent at 4 months (19% increased food intake in comparison to 36, 41, and 33% in animals at 1, 2, and 6 months, respectively). However, we did not observe changes in circulating levels of the orexigenic signal ghrelin. 1 week of cold exposure in ferrets also produced decreased adiposity (decreased size of interscapular, inguinal, and retroperitoneal adipose tissues), which was, however, not reflected as a decrease in body weight (Table 2B). One limitation of the ferret study is that we could not measure circulating leptin and ghrelin, due to a lack of ferret specific antibodies and reagents to detect these hormones.

**Table 2:** Body weight, adiposity, food intake and serum parameters.

**(A) WISTAR RATS (DIFFERENT AGES)**						
		**Body weight (g)**	**Adiposity (%)**	Food intake (Kcal/animal^*^day)	**Leptin (ng/ml)**	**Ghrelin (ng/ml)**
1 month	Control	86.2 ± 2.2	1.87 ± 0.06	38.8 ± 0.4	3.18 ± 0.18	13.5 ± 1.6
	Cold	72.8 ± 2.3^*^	1.14 ± 0.08^*^	52.6 ± 1.0^*^	1.59 ± 0.09^*^	11.2 ± 2.2
2 months	Control	199 ± 5	4.42 ± 0.32	56.2 ± 2.7	5.26 ± 0.51	21.5 ± 2.0
	Cold	196 ± 5	2.91 ± 0.30^*^	79.2 ± 2.4^*^	2.64 ± 0.27^*^	21.4 ± 3.4
4 months	Control	273 ± 6	5.89 ± 0.18	53.7 ± 3.1	7.13 ± 0.20	32.4 ± 12.2
	Cold	244 ± 7^*^	4.68 ± 0.46^*^	64.1 ± 1.5^*^	3.75 ± 0.18^*^	35.4 ± 12.8
6 months	Control	281 ± 6	7.44 ± 0.24	54.5 ± 1.0	8.04 ± 0.43	24.0 ± 1.6
	Cold	267 ± 6	5.42 ± 0.43^*^	72.6 ± 2.2^*^	3.79 ± 0.37^*^	18.4 ± 3.9
**(B) FERRETS (3 MONTHS OF AGE)**						
		**Body weight (g)**	**Interscapular AT (g)**	**Inguinal AT (g)**	**Retroperitoneal AT (g)**	**Mesenteric AT (g)**
	Control	623 ± 60	0.19 ± 0.04	2.15 ± 0.43	0.95 ± 0.20	3.34 ± 0.61
	Cold	510 ± 36	0.05 ± 0.02^*^	0.91 ± 0.24^*^	0.25 ± 0.12^*^	2.73 ± 0.67

**(A)** Body weight (g), adiposity (%), food intake (Kcal/animal^*^day), circulating leptin (ng/ml), and Ghrelin (ng/ml) in control and cold−exposed Wistar rats. **(B)** Body weight (g), and weight (g) of interscapular, inguinal, retroperitoneal, and mesenteric adipose tissues (AT) in control and cold−exposed ferrets. Results represent means ± SEM (n = 5−7).

* *Effect of cold exposure (cold−exposed animals vs. their respective controls; Student's t−test, p < 0.05, or indicated when different)*.

### Cold exposure regulation of key food intake control genes in the hypothalamus

**Figure 1:**
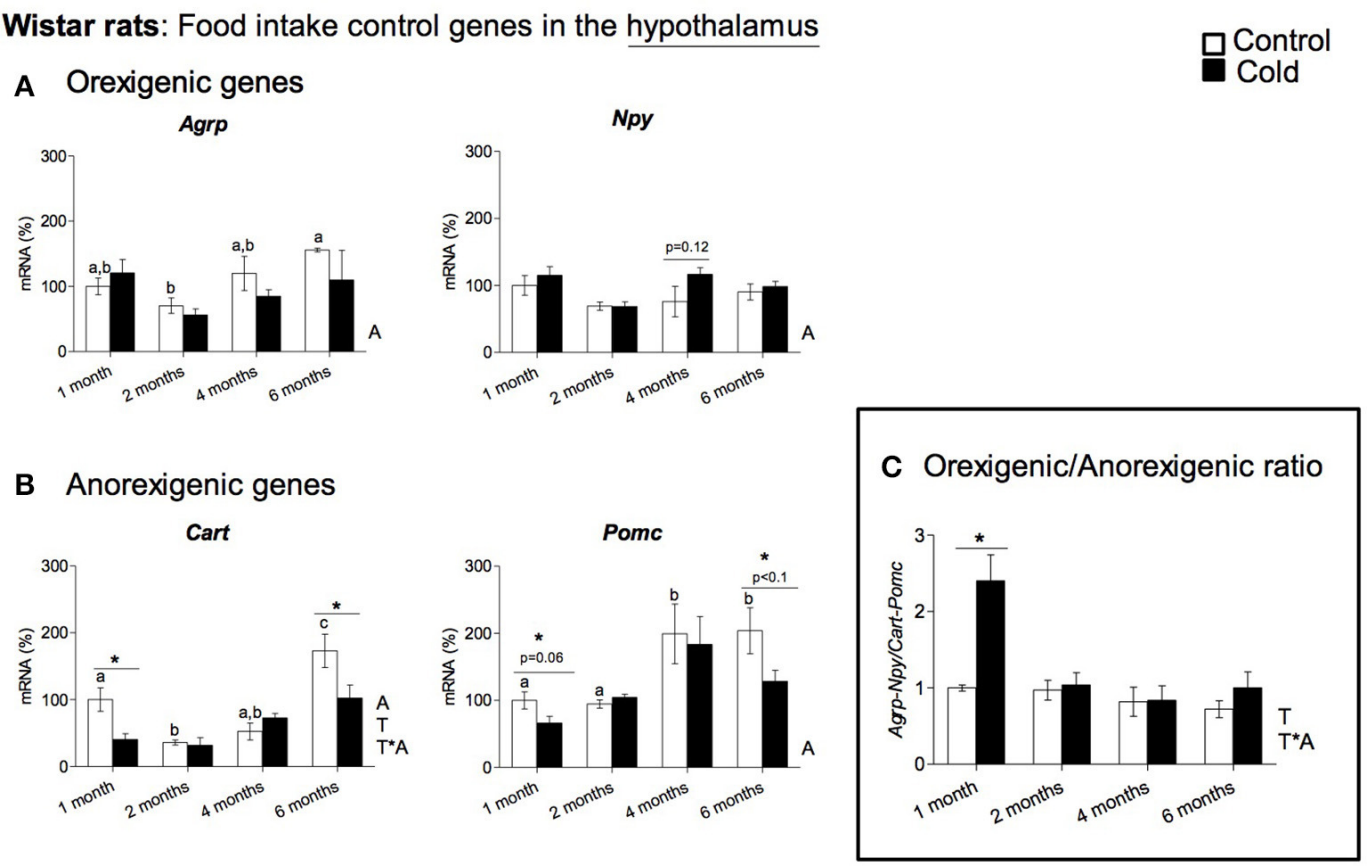
Gene expression of **(A)** orexigenic (*Agrp* and *Npy*) and **(B)** anorexigenic (*Cart* and *Pomc*) related genes measured by qRT-PCR in the hypothalamus of rats of different ages (from 1 to 6 months) housed at different room temperatures: 22°C (control) or 4°C for 1 week (cold). Results represent mean ± SEM (*n* = 5–6) of ratios of specific mRNA levels relative to *Gdi*, expressed as a percentage, where the 1-month control group was set as 100%. The orexigenic/anorexigenic ratio **(C)** was calculated as the sum of the expression of the orexigenic peptide (*Agrp* and *Npy*) genes divided by the sum of the expression of the orexigenic peptide (*Cart* and *Pomc*) genes. Bars not sharing common letters (a,b,c) are significantly different (one-way ANOVA, *p* < 0.05). DMS *post-hoc* was used after one-way ANOVA analysis. ^*^Effect of cold exposure (cold-exposed rats vs. their respective controls; Student's *t*-test, *p* < 0.05, or indicated when different). Two-way ANOVA (*p* < 0.05) was performed for cold and control conditions (T) and age (A). For this statistical analysis: T indicates effect of temperature, A effect of age, and T^*^A the interaction of temperature and age.

**Figure 2:**
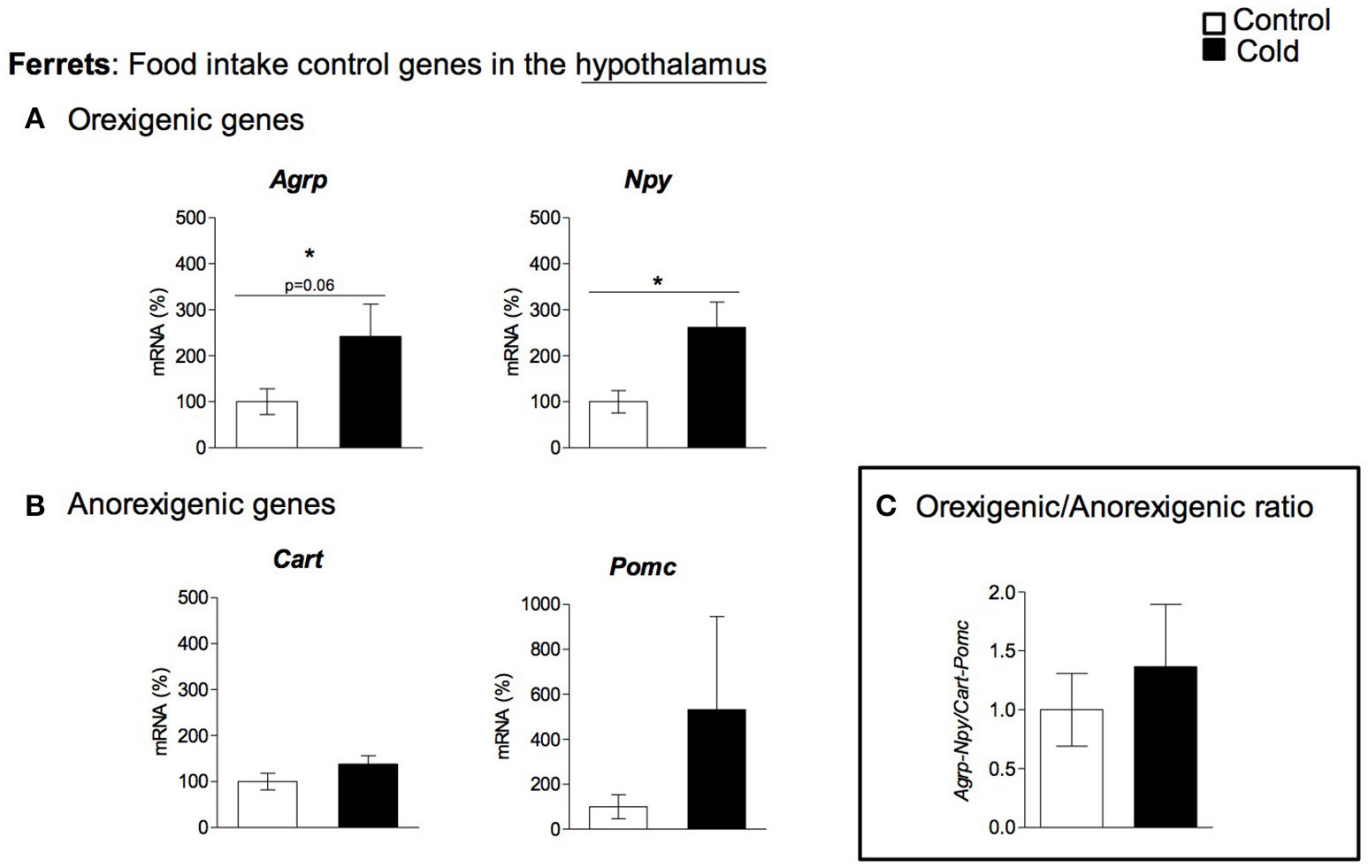
Gene expression of **(A)** orexigenic (*Agrp* and *Npy*) and **(B)** anorexigenic (*Cart* and *Pomc*) related genes measured by qRT-PCR in hypothalamus of adult ferrets housed at different room temperatures: 22°C (control) or 4°C for 1 week (cold). The orexigenic/anorexigenic ratio **(C)** was calculated as the sum of the expression of the orexigenic peptide (*Agrp* and *Npy*) genes divided by the sum of the expression of the orexigenic peptide (*Cart* and *Pomc*) genes. Results represent mean ± SEM (*n* = 5–7) of ratios of specific mRNA levels relative to *Rgp1*, expressed as a percentage of the value of fed animals in the Control group that was set to 100%. ^*^Effect of cold exposure (cold-exposed rats vs. their respective controls; Student's *t*-test, *p* < 0.05, or indicated when different).

In rats, cold exposure affected the gene expression of hypothalamic neuropeptide genes involved in food intake control, particularly of the anorexigenic genes *Cart* and *Pomc* (Figure 1A). *Cart* mRNA levels decreased in 1- and 6-month-old cold-exposed rats. The same tendency was observed for *Pomc*, although statistical significance was not reached (*p* = 0.06 and *p* = 0.08 Student's *t*-test in 1- and 6-month-old rats, respectively). However, no effect of cold exposure was observed for the orexigenic peptide genes studied, with the exception of a tendency to greater *Npy* mRNA levels at 4 months of age (*p* = 0.12, Student's *t*-test). Nevertheless, although orexigenic gene expression did not show a clear regulation by cold exposure, the orexigenic/anorexigenic gene expression ratio was highly increased in young animals, but no change was observed thereafter, in older rats (Figure 1C). These results reveal a clear interaction between the age of the animals and cold exposure response, which was evident in the expression of the anorexigenic gene *Cart* and in the orexigenic/anorexigenic gene expression ratio (two-way ANOVA, *p* < 0.05, Figures 1B,C). When considering 3-month-old ferrets, increased expression of the hypothalamic orexigenic genes *Npy* (*p* < 0.05, Student's *t*-test) and *Agrp* (*p* = 0.06, Student's *t*-test) was observed in response to cold exposure (Figure 2A). In this animal model, expression of the anorexigenic genes *Cart* and *Pomc* remained unchanged (Figure 2B) and no effect was observed in the orexigenic/anorexigenic gene expression ratio (Figure 2C).

### Cold exposure regulation of leptin signaling genes in the hypothalamus

**Figure 3:**
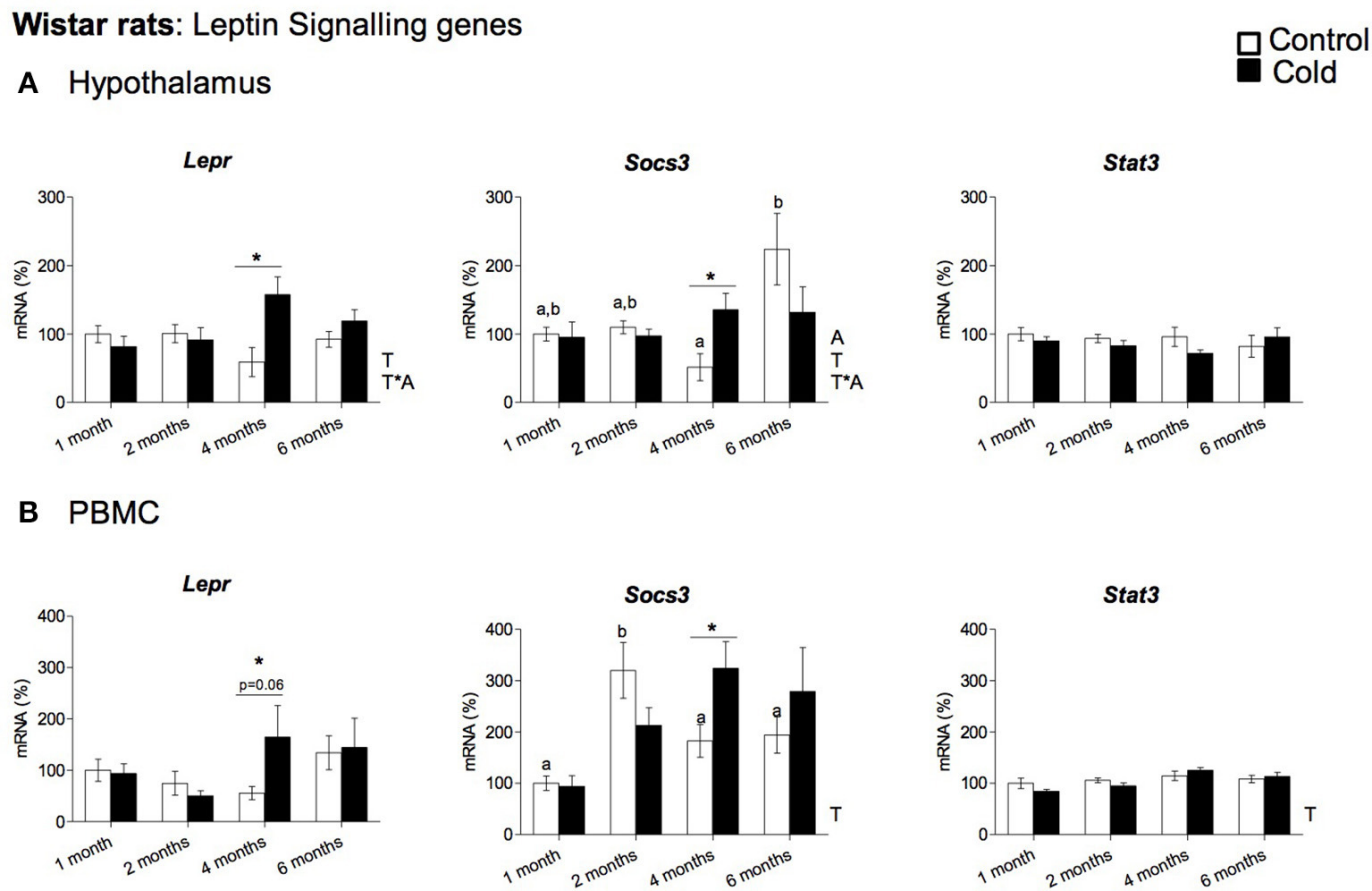
Gene expression of leptin signaling related genes (*Lepr, Socs3* and *Stat3*) measured by qRT-PCR in **(A)** hypothalamus and **(B)** PBMC of rats (from 1 to 6 months) housed at different room temperatures: 22°C (control) or 4°C for 1 week (cold). Results represent mean ± SEM (*n* = 5–6) of ratios of specific mRNA levels relative to *Gdi* for hypothalamus and *Lrp10* for PBMC, expressed as a percentage where the 1-month control group was set as 100%. Bars not sharing common letters (a,b) are significantly different (one-way ANOVA, *p* < 0.05). DMS *post-hoc* was used after one-way ANOVA analysis. ^*^Effect of cold exposure (cold-exposed rats vs. their respective controls; Student's *t*-test, p < 0.05, or indicated when different). Two-way ANOVA (*p* < 0.05) was performed for cold and control conditions (T) and age (A). For this statistical analysis: T indicates effect of temperature, A effect of age, and T ^*^A the interaction of temperature and age.

**Figure 4:**
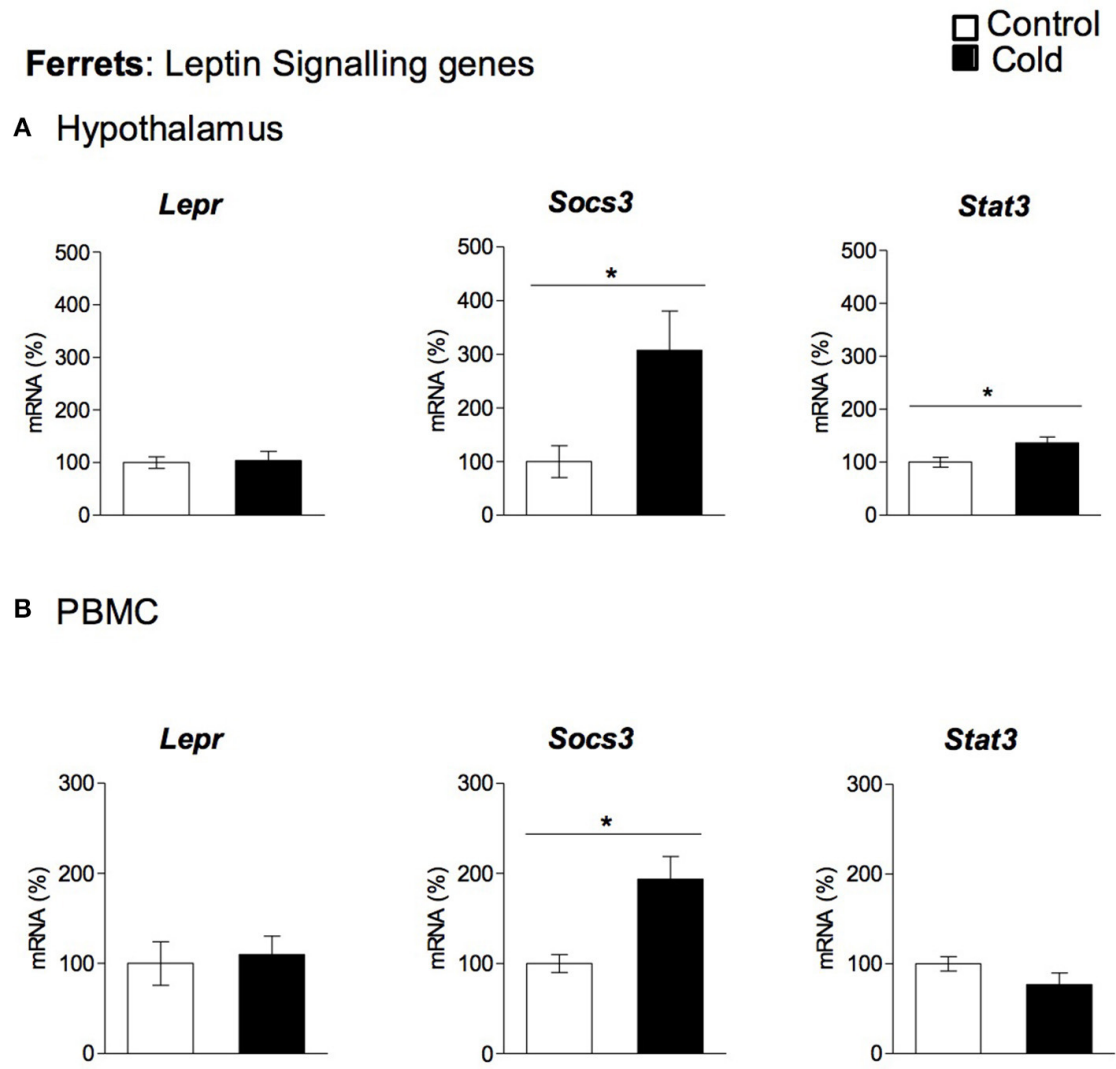
Gene expression of leptin signaling related genes (*Lepr, Socs3* and *Stat3*) measured by qRT-PCR in **(A)** hypothalamus and **(B)** PBMC of adult ferrets acclimatized to different room temperatures: 22°C (control) or 4°C for 1 week (cold). Results represent mean ± SEM (*n* = 5–7) of ratios of specific mRNA levels relative to *Rgp1* for hypothalamus, and *Mettl2a* for PBMC, expressed as a percentage of the value of fed animals in the Control group that was set to 100%. ^*^Effect of cold exposure (cold-exposed rats vs. their respective controls; Student's *t*-test, *p* < 0.05, or indicated when different).

Our data show that cold exposure induced changes in the expression of genes coding for key hypothalamic factors involved in leptin signaling, including those coding for LEPR, for SOCS3 and for the signal transducer and activator of transcription 3 (STAT3). In rats (Figure 3A), *Lepr* and *Socs3* mRNA levels were significantly elevated by cold exposure at the age of 4 months, showing an age-dependent cold exposure response (two-way ANOVA, *p* < 0.05). On the other hand, *Stat3* expression was not significantly altered by cold acclimation at any of the ages studied (Figure 3A). In ferrets, as seen in 4-month-old rats, we also observed increased *Socs3* mRNA levels in cold-exposed animals; in addition, *Stat3* gene expression was also increased, while gene expression of *Lepr* remained unchanged (Figure 4A).

### Regulation of key genes involved in food intake control and leptin signaling in PBMC by cold exposure

To evaluate whether PBMC reflect the cold exposure effects observed in the hypothalamus, we analyzed the gene expression of the key orexigenic and anorexigenic neuropeptides (*Agrp, Npy, Cart*, and *Pomc*) and the genes involved in leptin signaling (*Lepr, Socs3*, and *Stat3*) in these cells. In rats, PBMC expressed three of the orexigenic/anorexigenic genes studied: the orexigenic *Agrp* and *Npy* and the anorexigenic *Pomc*, while *Cart* mRNA did not reach detectable levels. No significant changes in gene expression were observed for any of these genes in response to cold exposure (data not shown). Interestingly, leptin signaling gene expression regulation in rat PBMC seems to reflect hypothalamic cold exposure regulation. As seen in the hypothalamus, *Lepr* and *Socs3* gene expression increased with cold exposure in the PBMC of 4-month-old animals, whereas *Stat3* expression was not significantly altered by cold acclimation at any of the ages studied (Figure 3B). In ferrets we did not detect quantifiable levels of the orexigenic (*Agrp, Npy)* or anorexigenic (*Cart, Pomc*) genes in PBMC, but these cells expressed the leptin signaling-related genes: *Stat3, Socs3*, and *Lepr* (Figure 4B). Interestingly, as seen in the hypothalamus of these animals, gene expression of *Socs3* was increased in PBMC as a result of cold exposure.

### Correlation analysis

As expected, results of the correlation analysis, performed in rats, showed a clear negative correlation between food intake and circulating leptin at the different ages studied; however, a positive correlation between the hypothalamic orexigenic/anorexigenic gene expression ratio and food intake, and a negative correlation with circulating leptin levels was only observed in the youngest animals, which were more sensitive to cold exposure (Table 3). Interestingly, when we analyzed correlations at the different ages studied we found significant relationships between circulating expression of leptin-signaling related genes and leptin and food intake in the hypothalamus and PBMC precisely at 4 months, the age at which we observed the effect of cold-exposure at gene expression level in our animals (see Table 3 for detailed correlations). Moreover, we found a strong, positive correlation (*p* ≤ 0.01) for *Socs3* between PBMC and the hypothalamus. No significant correlation for leptin signaling-related genes was observed between PBMC and the hypothalamus in ferrets (data not shown).

**Table 3:** Correlation analysis.

	**1 month**	**2 months**	**4 months**	**6 months**
**GENERAL PARAMETERS**				
Food intake−Hypothalamic orexigenic/anorexigenic ratio	*r =* 0.735	*r =* −0.036	*r =* 0.273	*r =* 0.454
	*p ≤* 0.01	*p =* 0.92	*p =* 0.48	*p =* 0.19
Leptin−Hypothalamic Orexigenic/Anorexigenic ratio	*r =* −0.653	*r =* 0.073	*r =* −0.026	*r =* −0.280
	*p =* 0.03	*p =* 0.84	*p =* 0.95	*p =* 0.43
Food intake−leptin	*r =* −0.923	*r =* −0.684	*r =* −0.873	*r =* −0.804
	*p ≤* 0.01	*p ≤* 0.01	*p ≤* 0.01	*p ≤* 0.01
**LEPTIN SIGNALING**−**RELATED GENES**				
**Hypothalamus**				
Food intake−*Lepr*	*r =* −0.330	*r =* −0.237	*r =* 0.621	*r =* 0.526
	*p =* 0.32	*p =* 0.51	*p =* 0.08	*p =* 0.08
Leptin−*Lepr*	*r =* 0.199	*r =* −0.079	*r =* −0.652	*r =* −0.186
	*p =* 0.56	*p =* 0.83	*p =* 0.06	*p =* 0.56
Food intake−*Socs3*	*r =* −0.169	*r =* −0.273	*r =* 0.721	*r =* −0.333
	*p =* 0.62	*p =* 0.42	*p =* 0.02	*p =* 0.29
Leptin−*Socs3*	*r =* 0.138	*r =* 0.208	*r =* −0.670	*r =* 0.305
	*p =* 0.69	*p =* 0.54	*p =* 0.03	*p =* 0.34
**PBMC**				
Food intake− *Lepr*	*r =* 0.010	*r =* −0.316	*r =* 0.919	*r =* −0.092
	*p =* 0.98	*p =* 0.37	*p ≤* 0.01	*p =* 0.83
Leptin−*Lepr*	*r =* 0.083	*r =* 0.020	*r =* 0.752	*r =* −0.121
	*p =* 0.82	*p =* 0.96	*p =* 0.03	*p =* 0.78
Food intake−*Socs3*	*r =* −0.224	*r =* −0.496	*r =* 0.491	*r =* 0.262
	*p =* 0.48	*p =* 0.10	*p =* 0.13	*p =* 0.44
Leptin−*Socs3*	*r =* 0.213	*r =* 0.234	*r =* −0.638	*r =* −0.329
	*p =* 0.51	*p =* 0.46	*p =* 0.04	*p =* 0.32
**Hypothalamus vs. PBMC**				
*Lepr*	*r =* 0.673	*r =* 0.085	*r =* 0.235	*r =* −0.192
	*p =* 0.03	*p =* 0.83	*p =* 0.61	*p =* 0.65
*Socs3*	*r =* 0.576	*r =* −0.128	*r =* 0.796	*r =* −0.330
	*p =* 0.06	*p =* 0.71	*p ≤* 0.01	*p =* 0.32

*Analysis of individual correlations between food intake, circulating leptin levels, and hypothalamic orexigenic/anorexigenic gene expression ratio; between food intake, circulating leptin levels, and leptin signaling−related gene expression in hypothalamus; and between Obrb and Socs3 mRNA expression in hypothalamus, and PBMC in rats of different ages. The orexigenic/anorexigenic gene expression ratio was calculated as the sum of the expression of the orexigenic peptide (Agrp and Npy) genes divided by the sum of the expression of the orexigenic peptide (Cart and Pomc) genes. mRNA levels were measured by qRT−PCR. Pearson's correlation indices (r) are given together with the threshold of significance (p-value). Data of both control and cold exposed rats were included for each age (a total of n = 5−6 animals per group). Shading indicates significant correlations*.

## Discussion

Prolonged cold exposure sympathetically stimulates energy expenditure by increasing basal metabolism and BAT thermogenesis (Himms-Hagen, 1990; Nedergaard et al., 2007; Reynés et al., 2015). To counteract increased energy expenditure, cold exposure also induces hyperphagia, (Mercer et al., 1997; Bing et al., 1998a,b; Park et al., 2007; Tang et al., 2009; Lau et al., 2016). The anorexigenic hormone leptin, mainly expressed in the adipose tissue, seems to play a key role in energy intake regulation during cold acclimation; however, the molecular mechanisms involved are not yet clear (Bing et al., 1998a,b; Ricci et al., 2000; Zhang and Wang, 2007; Tang et al., 2009). Moreover, as reported recently, in mice, leptin treatment induces brain pyrexic mechanisms to prevent heat loss during cold exposure, but does not directly affect the thermogenic process; thus, thermogenesis is unlikely to be involved in changes in body weight of cold-exposed animals (Fischer et al., 2016), giving more relevance to the potential effects of leptin affecting food intake control.

The results obtained here demonstrate that leptin levels in rats decrease after cold acclimation regardless of the age of animals, and show a strong negative correlation between circulating leptin levels and food intake, suggesting a critical role of this hormone in the hyperphagia observed after cold exposure. Another important hormone with a role in food intake is ghrelin, which is mainly expressed in the stomach and increases in conditions of negative energy balance (Wren et al., 2001). Contrary to what was expected (Tomasik et al., 2005), our study showed that circulating serum ghrelin levels were not affected by cold exposure at any of the ages studied.

The molecular effectors of leptin action in the hypothalamus are the anorexigenic peptides POMC and CART and the orexigenic peptides NPY and AGRP; however, their involvement in increased food intake in cold-exposed rodents is not clear, and most of the differences observed could be related to differences between rodent species and to the length and magnitude (°C) of cold exposure. For instance, while 28 day-cold exposure (4°C) increases *Agrp* gene expression in Brandt's voles (Tang et al., 2009), chronic (9–16 weeks) mild cold exposure (22°C vs. 30°C) in mice reduces *Cart* gene expression (Lau et al., 2016). Regarding NPY, different effects have been observed depending on the time of cold exposure (Mercer et al., 1997; Park et al., 2007; Tang et al., 2009; Lau et al., 2016). Our data show that the expression of genes coding for anorexigenic peptides, especially *Cart*, is decreased in the hypothalamus after 1 week of cold exposure in rats, as has been reported in mice (Lau et al., 2016). The differential expression of this gene is dependent on age, and was only observed in the youngest (1 month) and surprisingly in the oldest animals (6 months of age) studied. On the other hand, 1 week of cold exposure did not induce the expression of the orexigenic genes *Agrp* and *Npy* in rat hypothalamus, at any of the ages studied. These two neuropeptides have been accredited with sympathetic thermogenic suppression activity, and an absence of regulation may be interpreted as prevention of their hypothermic activity (Egawa et al., 1991; Ruan et al., 2014). Interestingly, when we analyzed a ratio computed for orexigenic vs. anorexigenic gene expression, we only found a high increase at the age of 1 month, suggesting that the neuronal adaptations to cold exposure are of special relevance in young animals. These results are consistent with our previous study with the same cohort of animals which show that cold acclimation for 1 week increases lipid metabolism, BAT thermogenesis, and WAT browning, especially in the youngest animals (Reynés et al., 2015). Thus, even if increased food intake were to be observed at different ages, our data suggest stronger hypothalamic adaptations at early ages to counteract grater energy expenditure.

In spite of the role of leptin in the gene expression regulation of the neuropeptides involved in food intake control, the results of effects of cold exposure on leptin signaling in rodents are controversial. While cold exposure in mice resulted in increased *Lepr* gene expression (Mercer et al., 1997), it did not result in alterations in the regulation of hypothalamic leptin signaling in Brandt's voles (Tang et al., 2009). Surprisingly, our rat study revealed a transient peak for *Socs3* mRNA expression in the hypothalamus of cold exposed animals only at 4 months of age, accompanied by increased mRNA expression levels of *Lepr*. Taking into account that SOCS3 is involved in the inhibition of leptin receptor signaling (Bjørbaek et al., 1999; Münzberg and Myers, 2005), the observed increase in *Lepr* mRNA expression could be a compensatory response to overcome the increased expression of *Socs3*. These results may be related to the smaller increase in food intake that is only observed in 4-month-old rats and suggest that specific factors may play a role in gene expression regulation at this particular age.

Most of the studies based on the hypothalamic contribution to food intake regulation by cold exposure are performed in rodents (Park et al., 2007; Lau et al., 2016). However, although extremely useful, rodents are not the best model to extrapolate cold exposure studies to a human situation (Bonet et al., 2013). This is because of the substantial contribution of the thermogenic activation of BAT to energy expenditure in rodents that is not the case in humans (Bonet et al., 2013). Thus, in addition to rodents, in the present study we newly used ferrets as an alternative approach to study the effects of cold exposure on the hypothalamus. We chose ferrets due to their similarities to humans in terms of adipose tissue organization and cold response (Murano et al., 2005; Fuster et al., 2009). In ferrets, *Cart* and *Pomc* mRNA levels remained stable, while both *Agrp* and *Npy* expression increased after 1 week of cold exposure (4°C). This was in contrast to the rat study, where gene expression changes were observed for anorexigenic genes, (mainly decreased expression of *Cart*), while expression of orexigenic genes (*Agrp* and *Npy*) was not affected. Other than the regulation of neuropeptide genes, hypothalamic leptin signaling was also modulated in ferrets as a result of cold acclimation. Interestingly, as observed in 4-month-old rats, we found increased *Socs3* expression. In this case, probably as a compensatory mechanism, we also observed increased expression of *Stat3*, which is a positive transducer of leptin signaling. A limitation of our work is that we were not able to measure food intake or circulating leptin levels in the ferret study; the former because food intake control was not the main aim of ferret experimental design, and the latter due to the lack of specific commercial kits. Moreover, our study was performed on the hypothalamus as a whole, not using specific hypothalamic regions, such as the arcuate nucleus that plays a key role in food intake control, which may mask some additional hypothalamic cold responses. Finally, we used animals of different sex (female and male animals in the rat and ferret experiments, respectively) because the data were generated from previous experimental designs. Thus, some of the differences between species observed in the regulatory pattern in hypothalamus and PBMC may be due to sex. However, this fact, apart from being a limitation of the experimental design, could be considered as a strength, because it gives extra translational value and robustness to those results which follow the same direction in the different animal models.

PBMC are increasingly being used for identification of practical molecular biomarkers. Here, we show that these cells can potentially be used as a surrogate tissue for hypothalamic studies. Rat PBMC expressed detectable levels of the hypothalamic neuropeptide genes *Npy, Pomc* and *Agrp*, but not *Cart*, as well as the key leptin signaling genes *Lepr, Socs3*, and *Stat3*. Remarkably, gene expression of these genes reflected the regulatory pattern observed in rat hypothalamus. As seen in the hypothalamus, *Npy, Pomc*, and *Agrp* gene expression was not affected by cold exposure at any of the ages studied, while the increased *Lepr* and *Socs3* gene expression observed in the hypothalamus of 4-month-old cold-exposed rats was seen in PBMC. In fact, a strong, positive correlation was found at this age between *Socs3* mRNA in PBMC and hypothalamus. Accordingly, in ferret PBMC, even though these cells did not express any of the hypothalamic neuropeptides studied, they reflected the increased *Socs3* mRNA levels observed in ferret hypothalamus. These results suggest that in both animal models PBMC are able to reproduce key effects of cold exposure on hypothalamic leptin signaling. The use of PBMC as a surrogate tissue to examine specific hypothalamic responses is supported by previous studies from our group. In particular, the correspondence of the *Npy* gene expression response in PBMC and hypothalamus to dietary interventions (Caimari et al., 2010a; Oliver et al., 2013) as well as weight gain and weight loss (Reynés et al., 2014) has been reported, validating the data of the present study.

## Conclusion

This work demonstrates that cold exposure induces an increase in orexigenic/anorexigenic gene expression ratio in rat hypothalamus, particularly at an early age (1-month-old animals), and mainly due to decreased expression of anorexigenic genes (*Cart* and *Pomc*). Despite the key role of leptin in neuropeptide regulation, expression of leptin signaling-related genes were affected by cold exposure only at the age of 4 months, indicating that some specific factors could affect leptin sensitivity at this particular age in rat hypothalamus. Furthermore, our study in adult ferrets revealed an increased expression of orexigenic peptide genes (*Agrp* and *Npy*), which could be related to hypothalamic food intake control. Moreover, cold-exposed ferrets also manifested increased hypothalamic expression of the leptin signaling related genes *Socs3* and *Stat3*. Finally, we feel that the most original and relevant data from the present research is that PBMC were able to reflect some of the characteristic hypothalamic gene expression features of cold exposure (mainly related to leptin signaling) observed in our study, both in rodents and ferrets. This suggests that these cells could be useful to perform studies aimed at the elucidation of hypothalamic mechanisms related to food intake control and energy metabolism regulation induced by exposure to low temperatures. Further studies to confirm the usefulness of PBMC to reflect hypothalamic responses, especially to cold exposure, are needed and can pave the way to faithfully use this easily obtainable biological material.

## Ethics statement

Bioethics Committee of the University of the Balearic Islands; approval: July 21, 2011.

## Author contributions

AP and PO conceived the experiment; BR, MKH, and EGR did most of the experimental work; BR, PO, JK, and AP wrote the manuscript; and all the authors participated in critical revising of the manuscript.

### Conflict of interest statement

The authors declare that the research was conducted in the absence of any commercial or financial relationships that could be construed as a potential conflict of interest.
